# Supplementary material for: Topological Anderson Insulator in Cation-Disordered Cu2ZnSnS4
Source: Nanomaterials (Basel). 2021 Oct 1;11(10):2595. doi: 10.3390/nano11102595 (PMC8540407; doi:10.3390/nano11102595)
Supplement: Supplementary file 1 [file nanomaterials-11-02595-s001.zip › nanomaterials-1342402-supplementary-materials.pdf]

nanomaterials

MDPI

Supplementary Materials

Topological Anderson Insulator in Cation-Disordered Cu_2ZnSnS_4

Binayak Mukherjee [1,*], Eleonora Isotta [1], Carlo Fanciulli [2], Narges Ataollahi [1] and Paolo Scardi [1,*]

[1] Department of Civil, Environmental and Mechanical Engineering, University of Trento, via Mesiano 77, 38123 Trento, Italy; eleonora.isotta@unitn.it (E.I.); narges.ataollahi@unitn.it (N.A.)
[2] National Research Council of Italy, Institute of Condensed Matter Chemistry and Technologies for Energy (CNR-ICMATE), Lecco Unit, via Previati 1/E, 23900 Lecco, Italy; carlo.fanciulli@cnr.it (C.F.)
* Correspondence: binayak.mukherjee@unitn.it (B.M.); paolo.scardi@unitn.it (P.S.)

Supplementary Note 1: Band Gap in Disordered CZTS

Despite appearances to the contrary in Figure 1f, disordered CZTS does in fact have a global band gap, albeit a complicated one. In our previous article, [25], we have experimentally measured the optical gap, which shows a large value of ~1.5 eV; notably however, the Tauc plot obtained from UV-vis spectroscopy shows large Urbach tailing, as seen in the image below (Figure S1a), Figure S3 from reference [25] (Isotta et al, Phys. Rev. Applied 14, 064073).

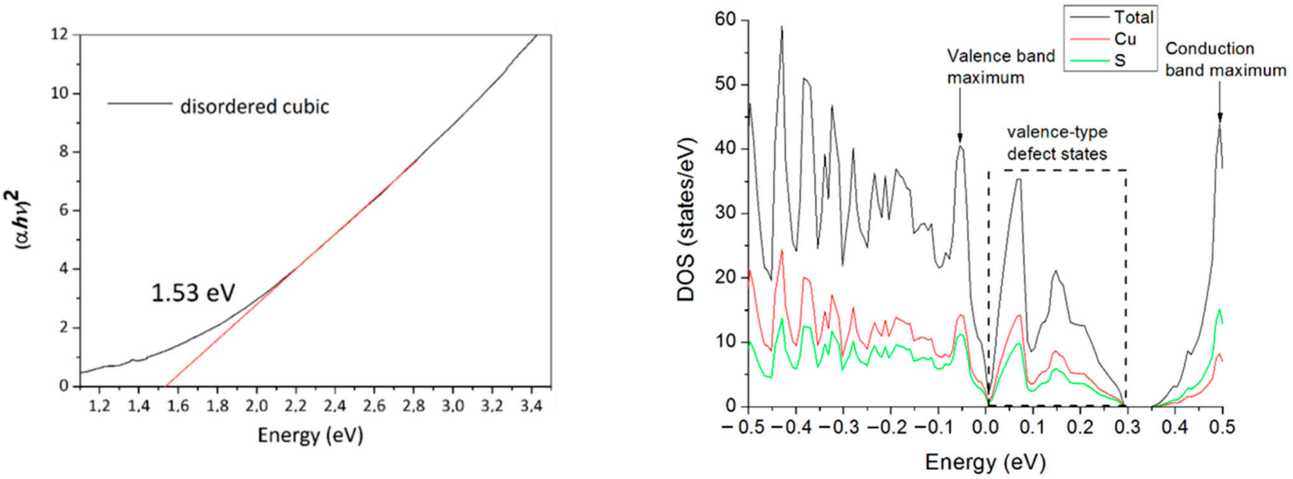

(**a**) (**b**)

Figure S1. The band gap of disordered CZTS seen from (**a**) Tauc-plot from UV-Visible spectroscopy; (**b**) density of states calculated using the SCAN meta-GGA functional.

The calculated density of states (above, right) shows that the valence band is dominated by Cu-d electrons, while the conduction band is predominantly composed of S-p electrons, with the presence of large valence type defect states just above the Fermi energy, which is set to 0. The large Urbach tailing in the Tauc plots can be accounted for by these defect states, which of course arise due to the highly disordered nature of the material. For a detailed theoretical discussion on these defect states and how they improve experimentally measured thermoelectric properties, we refer the reader to Ref 28. These defect states, which originate from the same orbitals as the valence band, but lie mostly above

the Fermi energy, also represent the valence band maximum seen in Figure 1f, with a smaller gap to the bottom of conduction band, allowing for band inversion. The fact that the Fermi energy lies within the valence band of course is characteristic of p-type semiconductors, and does *not* imply that the system has no global gap, but rather that it has band tailing and a reduced gap.

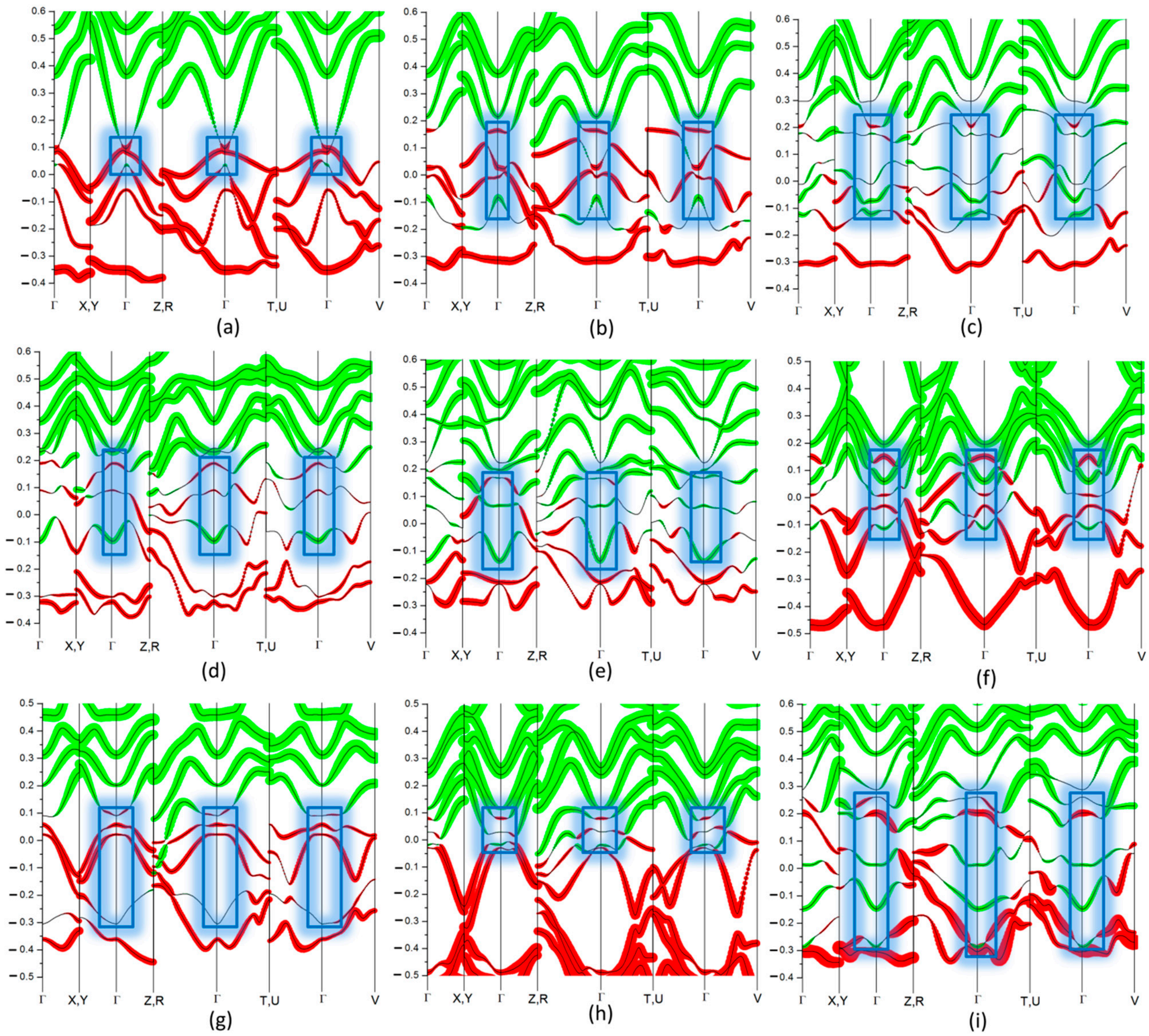

Figure S2. Bands for multiple configurations of disordered cubic CZTS. Symbols have save same meaning as in the main text.

Table S1. Total ground state energies of the multiple configurations of disordered cubic CZTS.

Configuration	Total energy
Config1 (Figure 1f)	−0.25723222 × 10^3
Config2 (Figure S1a)	−0.25487437 × 10^3
Config3 (Figure S1b)	−0.25700924 × 10^3
Config4 (Figure S1c)	−0.25462498 × 10^3
Config5 (Figure S1d)	−0.25416696 × 10^3
Config6 (Figure S1e)	−0.25364071 × 10^3
Config7 (Figure S1f)	−0.25547144 × 10^3
Config8 (Figure S1g)	−0.25522658 × 10^3
Config9 (Figure S1h)	−0.25509927 × 10^3
Config10 (Figure S1i)	−0.25400458 × 10^3

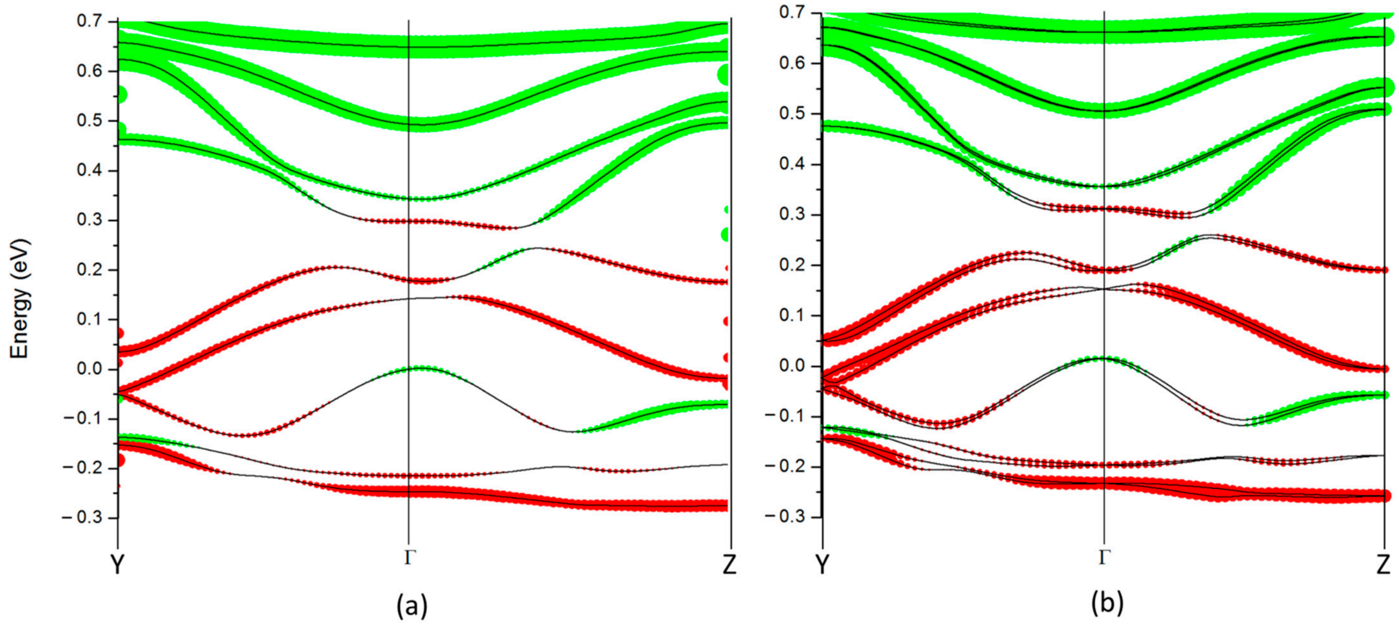

Figure S3. Comparison of bands for disordered cubic CZTS (**a**) without and (**b**) with spin-orbit coupling. Symbols have save same meaning as in the main text.

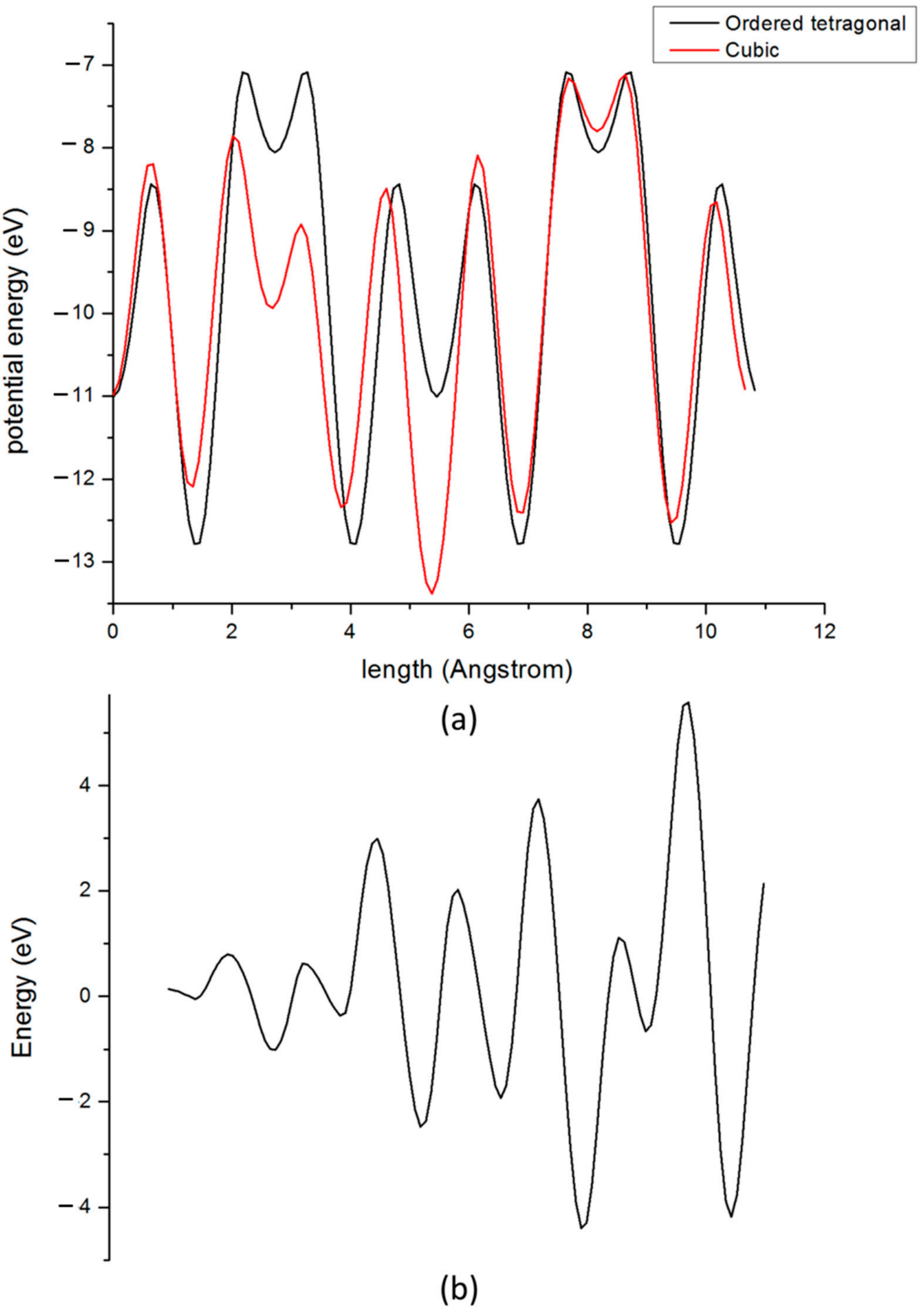

Figure S4. (**a**) Local potential in the Z-direction for ordered tetragonal (black) and disordered cubic (red) CZTS; (**b**) Difference between the potentials. The lattice mismatch in the Z-direction between the tetragonal and cubic supercells exaggerates the difference.

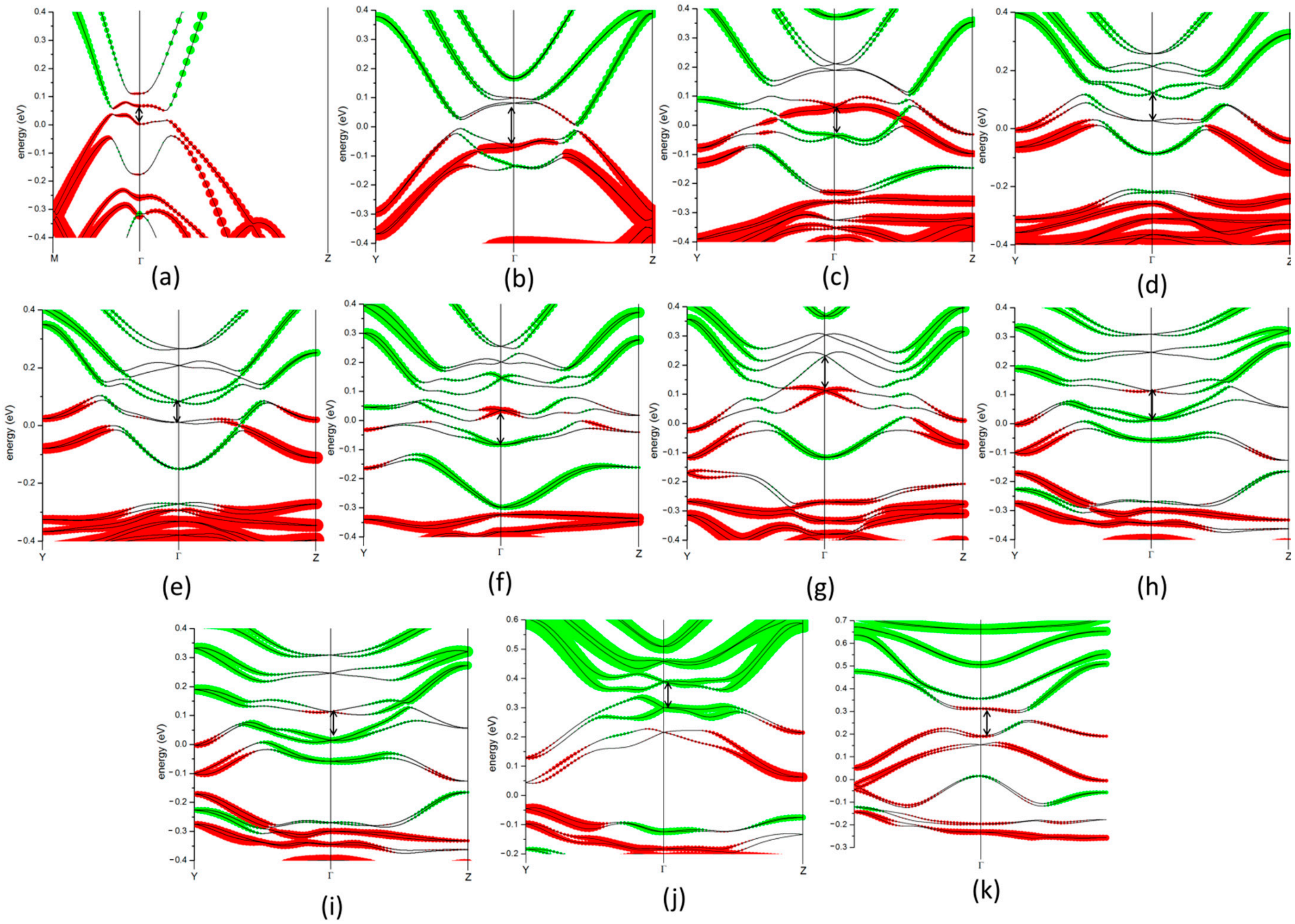

Figure S5. Adiabatic transition from (**a**) stannite CZTSe through (**k**) cubic CZTS. Intermediate images show the bands for intermediate states, each corresponding to a single interchange in cation positions. Arrows highlight the open inverted band gap.

Table S2. Total ground state energies for the intermediate configurations in the adiabatic transition from ordered to disordered CZTSe.

Step	Total ground state energy
Step 1 (Figure S4a)	-0.23556786×10^3
Step 2 (Figure S4b)	-0.23552479×10^3
Step 3 (Figure S4c)	-0.23484019×10^3
Step 4 (Figure S4d)	-0.23303846×10^3
Step 5 (Figure S4e)	-0.23233460×10^3
Step 6 (Figure S4f)	-0.23221546×10^3
Step 7 (Figure S4g)	-0.23220398×10^3
Step 8 (Figure S4h)	-0.23231033×10^3
Step 9 (Figure S4i)	-0.23122961×10^3
Step 10 (Figure S4j)	-0.23132112×10^3

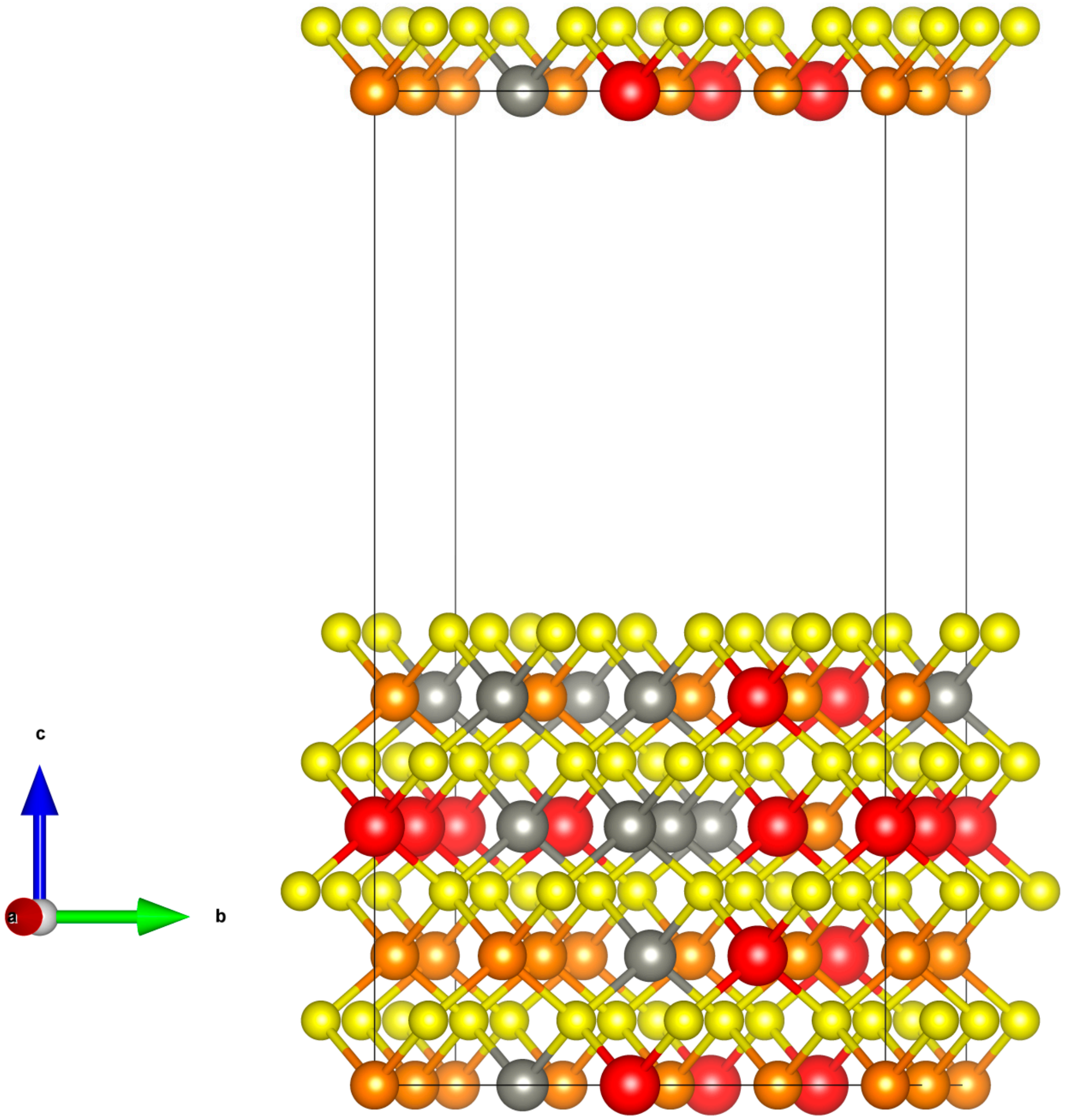

Figure S6. Surface slab geometry for the 001 surface of cubic CZTS with S termination. Orange atoms are Cu, grey atoms are Zn, red atoms are Sn and yellow atoms are S.

Supplementary Note 2: Grain Size Dependence of Mobility

Conclusive evidence in favor of a TI can be obtained via a direct experimental observation of the topological surface states with angle resolved photoemission spectroscopy (ARPES). However, this technique is suitable only for large single crystals with perfectly clean surfaces. Disordered cubic CZTS, synthesized as it is via reactive ball-milling, exists essentially in a metastable, nano-polycrystalline form, and is therefore unavailable for ARPES. Under such circumstances, transport experiments can provide an alternate route to support the presence of the surface states: given a sufficiently insulating bulk, a substantial contribution to conduction is expected from the surface[1]. This feature can be verified by observing the grain size dependence of the electrical transport[2–5]. In particular, this should be seen as an increase in carrier mobility with a reduction of grain size, as a consequence of the topological surface states.

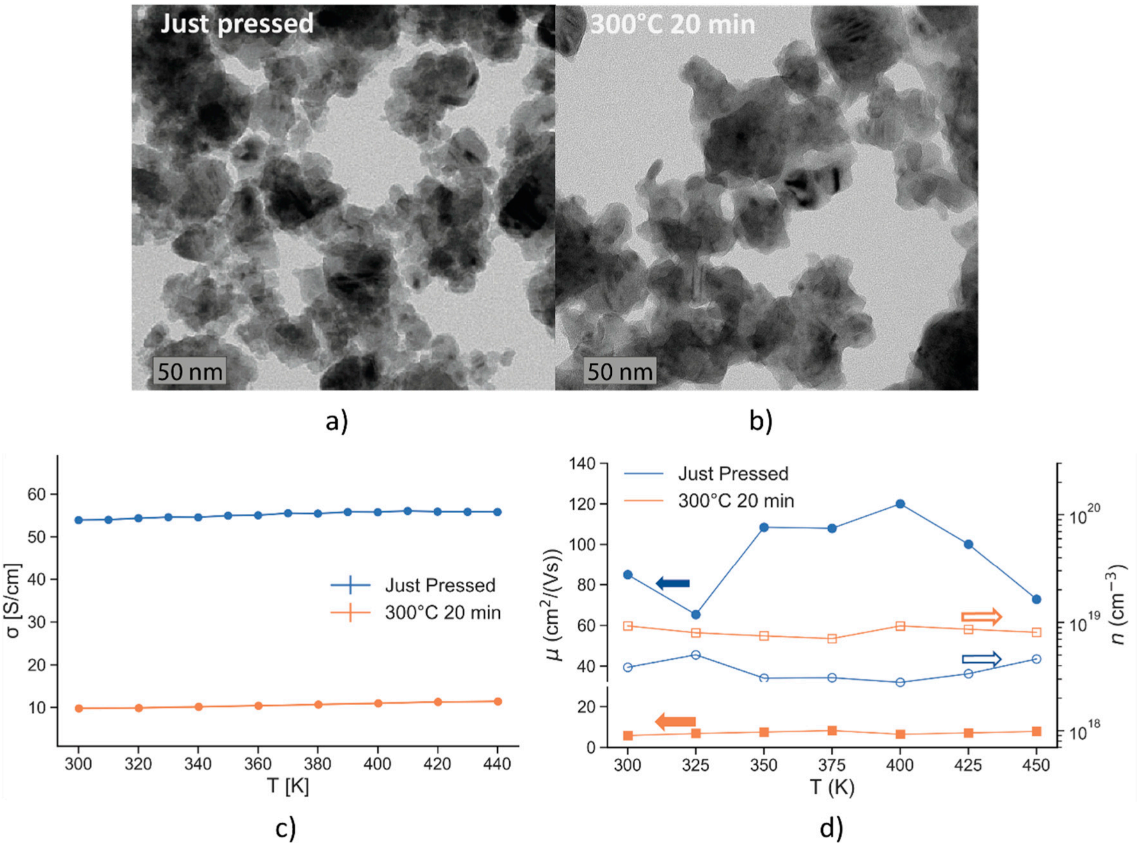

Figure S7. TEM imaging (**a**,**b**) and electrical transport measurements (**c**,**d**) for a just pressed and a thermally treated sample. The smaller grain size observed for the just pressed sample (in panel a and confirmed by XRD, se supplementary material for details) leads to a higher grain surface area which is thought at the origin of the increased electrical conductivity σ (**c**). Carrier concentration n and mobility μ measurements (**d**) demonstrate that the enhancement in σ is coming from a remarkable increase in carrier mobility, while carrier concentration remains in the same order of magnitude (considering the precision of Hall effect measurements). This atypical trend of properties might be due to scattering-resistant transport arising from the topological surface states.

As can be seen in Figure S6, the experimental measurements do indeed point to a significant surface contribution to conduction. Two cubic samples have been compared: one has been obtained just with a cold-pressing step, while the other has been thermally treated at 573K. This thermal treatment is performed to promote some grain growth, while keeping the sample below the cubic to tetragonal phase transition (at ~673K). This grain growth is confirmed through TEM imaging (Figure S6a, b) and X-ray Diffraction (with, Supplementary Figure S6). By modelling the XRD data through the WPPM approach[6], we estimate the coherent domain size for the just pressed and thermally treated samples as 9.7 nm and 13.4 nm respectively. . Electrical conductivity is found higher for the sample showing smaller grains (figure S6c). This is in contradiction to a general trend in semiconductors[7]: thermal treatments typically lead to higher density, improved connectivity between adjacent domains, and a reduction in carrier scattering from the grain boundary, all of which should improve electrical conduction. In order to identify the origin of this atypical trend in conductivity, we performed mobility and carrier concentration measurements. The carrier concentration is found to be in the same order of magnitude for both the samples (Figure S6d, right axis), with the improved conductivity of the just pressed sample (with smaller grains) originating from an enhancement of carrier mobility (Figure S6d, left axis). This is what would be expected from a higher surface area characterized by scattering-resistant charge transport arising from the topological surface states.

Several sample differences might possibly contribute to the observed variation of transport properties between the samples with different grain size. For example, thermal

treatments are expected to trigger some cation ordering. As such, the just pressed sample, by virtue of not being exposed to temperatures above RT, is most probably characterized by a higher cation disorder. However, in a recent study by some of the present authors, it has been demonstrated that cation disorder in CZTS seems to improve conductivity by introducing additional gap states, thereby promoting p-type carrier concentration[8]. The carrier mobility, on the other hand, was typically found to get depressed with disorder, due to band flattening and increased scattering. This is in direct opposition to our current trends, where the more disordered just-pressed sample shows significantly higher mobility values. Another possible contribution might be from the surface chemistry, which may vary from that of the bulk, although we would expect the changes to be consistent for both the samples. As such, the inverse correlation between mobility and grain size can be best explained by the higher surface-to-volume ratio in the smaller grains, and the correspondingly greater abundance of the topological surface states [1] predicted from theory. These robust surface states would allow charge carriers to skip around defects at the grain boundary, enabling carrier transport without backscattering, and explaining the experimentally observed increase in mobility in the just pressed sample with smaller grains.

While the experimental evidence provided above falls somewhat short of being sufficient for conclusive proof, it is nevertheless definitely in agreement with the results of the DFT calculations. As such, we can consider it to be satisfying a necessary condition for a topological insulator phase.

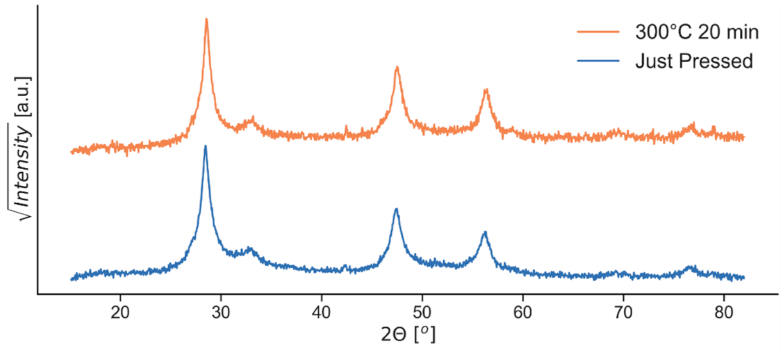

(a)

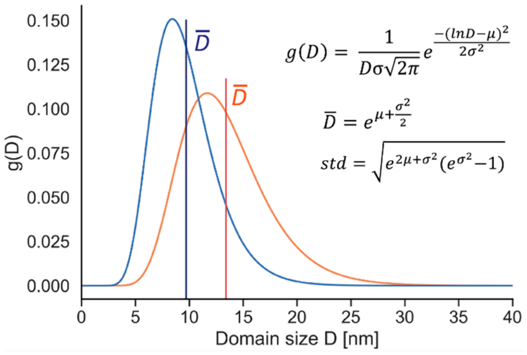

(b)

	$\bar{D}$ [nm]	std [nm]
**Just pressed**	9.7(3)	3(1)
**300°C 20 min**	13.4(8)	4.1(2)

(c)

Figure S8. (**a**) XRD patterns for a just pressed and a thermally treated sample of cubic CZTS. The purpose of the thermal treatment was to increase the domain size while keeping the phase cubic. Rietveld refinement with the software TOPAS was performed on XRD data using the WPPM model for size broadening. This assumes a lognormal distribution of domain sizes, reported in panel (**b**) for both the samples. Equations from top to bottom represent a lognormal distribution, the arithmetic mean domain size and the corresponding standard deviation. The thermal treatment leads to a broader and right-shifted distribution, indicating some grain growth, in accordance with TEM observations reported in the main text. Distribution parameters are visible in Table (**c**).

References

1. Ando, Y. Topological Insulator Materials. *J. Phys. Soc. Jpn.* **2013**, *82*, 1–32, doi:10.7566/jpsj.82.102001.
2. Taskin, A.A.; Ren, Z.; Sasaki, S.; Segawa, K.; Ando, Y. Observation of Dirac Holes and Electrons in a Topological Insulator. *Phys. Rev. Lett.* **2011**, *107*, 016801, doi:10.1103/physrevlett.107.016801.
3. Zhang, X.; Butch, N.P.; Syers, P.; Ziemak, S.; Greene, R.L.; Paglione, J. Hybridization, Inter-Ion Correlation, and Surface States in the Kondo Insulator SmB_6. *Phys. Rev. X* **2013**, *3*, 1–7, doi:10.1103/physrevx.3.011011.
4. Kim, D.J.; Thomas, S.; Grant, T.; Botimer, J.; Fisk, Z.; Xia, J. Surface Hall Effect and Nonlocal Transport in SmB_6: Evidence for Surface Conduction. *Sci. Rep.* **2013**, *3*, 3150, doi:10.1038/srep03150.
5. Wolgast, S.; Kurdak, Çağlıyan; Sun, K.; Allen, J.W.; Kim, D.-J.; Fisk, Z. Low-temperature surface conduction in the Kondo insulator SmB_6. *Phys. Rev. B* **2013**, *88*, 1–5, doi:10.1103/physrevb.88.180405.
6. Scardi, P.; Ricardo, C.L.A.; Perez-Demydenko, C.; Coelho, A.A. Whole powder pattern modelling macros for TOPAS. *J. Appl. Crystallogr.* **2018**, *51*, 1752–1765, doi:10.1107/s160057671801289x.
7. Kuo, J.J.; Kang, S.D.; Imasato, K.; Tamaki, H.; Ohno, S.; Kanno, T.; Snyder, G.J. Grain boundary dominated charge transport in Mg_3Sb_2-based compounds. *Energy Environ. Sci.* **2018**, *11*, 429–434, doi:10.1039/c7ee03326e.
8. Isotta, E.; Mukherjee, B.; Fanciulli, C.; Ataollahi, N.; Sergueev, I.; Stankov, S.; Edla, R.; Pugno, N.M.; Scardi, P. Origin of a Simultaneous Suppression of Thermal Conductivity and Increase of Electrical Conductivity and Seebeck Coefficient in Disordered Cubic Cu_2ZnSnS_4. *Phys. Rev. Appl.* **2020**, *14*, 064073, doi:10.1103/physrevapplied.14.064073.
